# Exploring the impact of age on the predictive power of the National Early Warning score (NEWS) 2, and long-term prognosis among patients reviewed by a Rapid Response Team: A prospective, multi-centre study

**DOI:** 10.1016/j.resplu.2024.100839

**Published:** 2024-12-10

**Authors:** Anna Thorén, Mikael Andersson Franko, Eva Joelsson-Alm, Araz Rawshani, Thomas Kahan, Johan Engdahl, Martin Jonsson, Therese Djärv, Martin Spångfors

**Affiliations:** a Department of Medicine Solna, Centre for Resuscitation Science, Karolinska Institutet, SE-171 77, Stockholm, Sweden; b Department of Clinical Physiology, Danderyd University Hospital, SE-182 88, Stockholm, Sweden; c Department of Clinical Science and Education, Södersjukhuset, Karolinska Institutet, SE-118 83, Stockholm, Sweden; d Department of Clinical Science and Education, Södersjukhuset, Centre for Resuscitation Science, Karolinska Institutet, SE-118 83, Stockholm, Sweden; e Department of Anaesthesia and Intensive Care, Södersjukhuset, SE-118 83, Stockholm, Sweden; f Department of Molecular and Clinical Medicine, Institute of Medicine, Wallenberg Laboratory, University of Gothenburg, SE-413 45, Gothenburg, Sweden; g Department of Cardiology, Sahlgrenska University Hospital/Mölndal, SE-413 45, Gothenburg, Sweden; h Division of Cardiovascular Medicine, Department of Clinical Sciences, Danderyd Hospital, Karolinska Institutet, SE-182 88, Stockholm, Sweden; i Department of Cardiology, Danderyd University Hospital, SE-182 88, Stockholm, Sweden; j Department of Acute and Reparative Medicine, Karolinska University Hospital, SE- 171 64, Stockholm, Sweden; k Department of Clinical Sciences, Anaesthesiology and Intensive Care, Lund University, SE-221 84, Lund, Sweden; l Department of Anaesthesia and Intensive Care, Kristianstad Hospital, SE-291 89, Kristianstad, Sweden

## Abstract

**Aim:**

To explore the impact of age on the discriminative ability of the National Early Warning Score (NEWS) 2 in prediction of unanticipated Intensive Care Unit (ICU) admission, in-hospital cardiac arrest (IHCA) and mortality within 24 hours of Rapid Response Team (RRT) review. Furthermore, to investigate 30- and 90-day mortality, and the discriminative ability of NEWS 2 in prediction of long-term mortality among RRT-reviewed patients.

**Methods:**

Prospective, multi-centre study based on 830 complete cases. Data was collected by RRTs in 24 hospitals between October 2019, and January 2020. All NEWS 2 scores were uniformly calculated by the study team. Age was analysed as a continuous variable, in a spline regression model, and categorized into five different models, subsequently explored as additive variables to NEWS 2. The discriminative ability of NEWS 2 was evaluated using the Area under the receiver operating characteristics (AUROC).

**Results:**

The discriminative ability of NEWS 2 alone in predicting 30-day mortality was weak. Adding age as a covariate improved the predictive performance (AUROC 0.66, 0.62–0.70 to 0.70, 0.65–0.73, *p* = 0.01, 95 % Confidence Interval). There were differences across age groups, with the best discriminative ability identified among patients aged 45-54 years. The 30- and 90-day mortality was 31% and 33% respectively.

**Results:**

Adding age as a covariate improved the discriminative ability of NEWS 2 in the prediction of 30-day mortality among RRT-reviewed patients, with variations observed across age categories. The long- term prognosis of RRT-reviewed patients was poor.

## Introduction

Many hospitalized patients are affected by significant adverse medical events such as unanticipated Intensive Care Unit (ICU) admission, in-hospital cardiac arrest (IHCA) and in-hospital death. About 60–84 % of these significant adverse medical events are preceded by prolonged periods of deviating vital signs,^1^, ^2^ and thus potentially avoidable.^3^

Rapid Response Systems were designed to facilitate early identification of patients at risk of deterioration and initiation of a timely clinical response,^4^, ^5^, ^6^ consisting of an afferent limb (identification and monitoring of patients, e.g., Early warning scores (EWS)) and an efferent limb (the clinical response, e.g., Rapid Response Teams (RRTs)).

The National Early Warning Score (NEWS), developed in 2012 and subsequently revised to NEWS 2 in 2017, has been extensively validated and shown superior to other EWS in identifying patients at risk of significant adverse medical events within 24 h.^7^, ^8^, ^9^ However, NEWS 2 is a “one size fits all” scoring model, and there are indications that NEWS 2 requires adjustments for specific conditions and categories of patients.^10^

Old patients constitute a significant proportion of the acutely ill admitted to hospitals.^11^ It is well established that age affects the mortality associated with vital sign abnormalities, thus being an important component of scoring systems aiming to predict mortality in specified groups of patients.^12^, ^13^, ^14^ Vital signs are also known to vary with increasing age. Hence, old patients may be a group who might benefit from an individual adjustment of NEWS 2, and it has been suggested that the inclusion of age could be advantageous in improving the performance of EWSs.^14^ Combining NEWS with age in an Emergency Department setting has recently shown to improve the discriminative ability in predicting mortality.^15^

Patients reviewed by RRTs constitute a high-risk population; within 24 h of review 25–36 % of the patients are admitted to the ICU, 7–10 % are dead and 1–2 % suffer from IHCA.^16^, ^17^ Hence, the decision process regarding ICU admission is of vital importance, yet challenging and influenced by multiple factors including ICU bed availability.^18^ Transfer to ICU may also be under influence by several forms of bias.^19^ An easy and user-friendly tool to serve as decision support would be valuable in this process. Until today, no such tools are available. Furthermore, previous studies have identified a knowledge gap regarding long- term mortality among patients reviewed by RRTs, as most previous studies have focused on short-term mortality.^16^, ^20^

Therefore, we aimed to:

i Explore the impact of age on the predictive performance of NEWS 2 in identifying patients at risk of unanticipated ICU, IHCA or in-hospital mortality within 24 h of RRT-review.
ii Investigate 30- and 90- day mortality among RRT-reviewed patients.
iii Explore if further development of NEWS 2 by adding age as a variable can improve the ability to predict long-term mortality among RRT-reviewed patients.

## Methods

### Study design and setting

Consecutive patients were included between October 2019, and January 2020, in this prospective, multi-centre, observational study.^17^ Swedish hospitals with an implemented RRT and NEWS/NEWS2 were eligible for participation. In all, 24 hospitals (university, county, and district) with varying geographical location participated (Table A 1).

All adult patients (≥18 years old) reviewed by the RRTs during the study period were eligible for inclusion. Patients with a decision on Limitations of medical treatment consisting of “no ICU care” prior to RRT-review, RRT-reviews performed as planned follow-up after ICU discharge, and pregnant women (including the postpartum period, i.e., 6 weeks following childbirth) were excluded.

Data on the clinical affiliation of the patient, symptoms/diagnosis at admission, the primary reason for RRT call, all vital signs required for the calculation of NEWS 2 scores (including information on hypercapnic failure, new-onset confusion, and use of oxygen) was collected by the RRTs. 10.13039/100014337Furthermore, decisions regarding level of care and an eventual new Limitation of medical treatment were recorded. Since miscalculation is a well-established source of error when using EWS, all NEWS 2 scores were uniformly calculated by the study team when analysing data.^8^, ^21^

After 24 h or more, information on ICU admission, IHCA and mortality was collected. Furthermore, an additional retrospective follow-up on long-term survival was performed approximately 21 months after the inclusion period, where data was collected from either medical records or the “Personal information directory”, containing population registration data. All registrations were performed in a secure, online system.

The study complies with the declaration of Helsinki and was approved by the Swedish Ethical Review Authority (2019–04269 and 2023–00187-02), which waived the need for informed consent. The reporting of the study was conducted in accordance with the Transparent Reporting of a multivariable prediction model for Individual Prognosis Or Diagnosis (TRIPOD) checklist for model development.^22^

### Definitions

The NEWS 2 is a compound score consisting of the physiological vital sign parameters respiration rate, oxygen saturation, systolic blood pressure, pulse rate, level of consciousness or new onset of confusion and body temperature. Each physiological parameter is scored from 0 to 3 points, related to an eventual deviation from the normal value. A binary score of two points is added for any use of supplemental oxygen, before adding up a final summed score. Furthermore, there is a dedicated oxygen saturation scale (Scale 2) for use in patients with hypercapnic, respiratory failure. There is also a clinical response scale linked to the NEWS 2 concept.

A NEWS 2 score of ≥ 5 points is defined as a key threshold, indicative of a medium risk of significant adverse medical event. Furthermore, a total score of ≥ 7 points is defined as a key trigger threshold, prompting an emergency assessment by a team with competence in critical care such as the RRT.

Age was explored as an additive variable to NEWS 2 by categorizing it according to two well-established ICU scoring models developed as severity-of-disease classification systems and risk prediction tools; the Acute Physiology, Age and Chronic Health Evaluation system (APACHE) 2, designed to predict mortality in the ICU,^23^ and the Simplified Acute Physiology Score (SAPS) 3, originally designed to predict in-hospital mortality, but in clinical practice in Sweden, it is used for prediction of 30-day mortality.^24^ Furthermore, we explored the age categories as previously stated by Smith et al.^14^

The different age models are described in Table A 2. We used the APACHE 2 categories and age categories as previously defined by Smith et al.^14^ Furthermore, we used the original SAPS 3 age category, and two modified versions of this model, the SAPS 3a and SAPS 3b (Table A 2).

As to the distribution of NEWS 2 score within the different age categories, the scores were weighted in proportion to the frequency of the specific score.

### Outcomes

The primary outcome was 30-day mortality. Secondary outcomes were unanticipated ICU-admission, IHCA, mortality, all within 24 h of RRT-review, the composite of these three outcomes, and 90-day mortality.

### Statistical analysis

Categorical variables were presented as numbers and percentages, and continuous variables as medians and interquartile ranges (Q1, Q3). Logistic regression was used for prediction of outcomes. We also used General Additive Models (GAM) with spline components for age and NEWS 2 when investigating non-linear associations.

Furthermore, the Area under the Receiver Operator Characteristic curves (AUROC) was used to evaluate the discriminative ability of NEWS 2 and age in identifying patients at risk of unanticipated ICU admission, IHCA, mortality or the composite of these three outcomes. Model calibration was evaluated with Brier scores, which measures the mean squared error of predicted probabilities. P-values and 95 % Confidence Intervals of Brier score differences were calculated using bootstrap sampling with 10,000 samples. Differences in AUROC between the models were compared by the DeLong test.

All tests were two-sided with a significance level of p < 0.05. All statistical analysis were performed with R, version 4.2.1, and IBM SPSS Inc., version 29 (Chicago, IL, USA).

## Results

### Patient characteristics

**Fig. 1:**
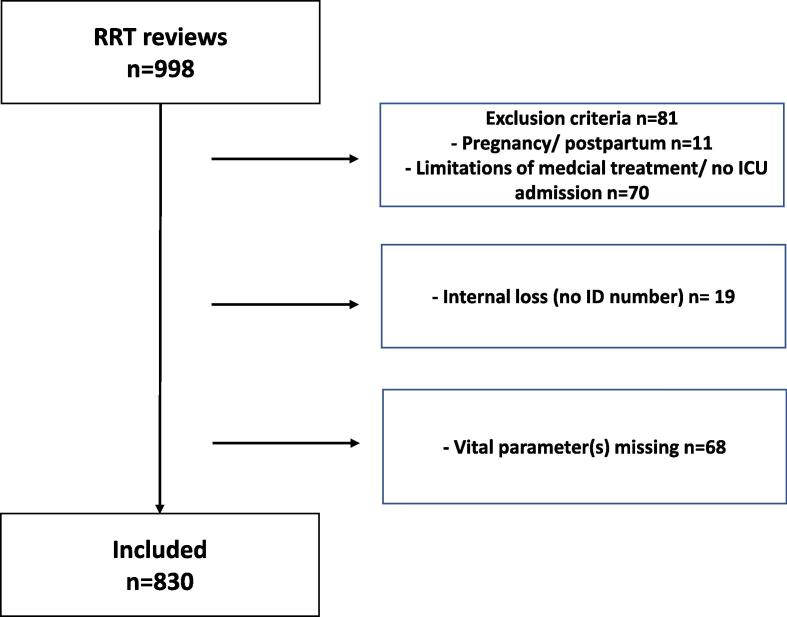
**Study cohort**. RRT, Rapid Response Team, ICU, Intensive Care Unit.

A total of 998 RRT-reviews were performed in the 24 participating hospitals. After applying the exclusion criteria, 917 patients remained (Fig. 1). Furthermore, 19 patients (1,9%) were excluded due to lack of personal ID number, which made follow-up impossible. In 68 patients (6,8%), there was at least one vital parameter missing, hence they were excluded. In all, 830 complete case patients were included in the analyses (Fig. 1).

The median age was 72 years, and 43 % of the patients were female (Table 1). The age distribution is illustrated in Figure A 1. The dominating location in hospital was medical wards (37 %), followed by surgical (26 %) and orthopaedic wards (10 %) (Table 1). Symptoms/diagnosis at admission is presented in Table 1 and Table A 3. An elevated NEWS 2 score was the most frequent RRT trigger (59 %), followed by staff concern (26 %) and other causes (15 %) (Table 1, Table A 3). In all, 108 patients (13 %) had a Limitation of medical treatment prior to RRT-review (DNACPR 12 %, other 1 %). The median NEWS 2 score at the time of RRT-review was 9 (Interquartile ranges 6, 11). A total NEWS 2 score of ≥ 7 was calculated in 74 % of the patients (Table 1). The median NEWS 2 scores of the five age categories are presented in Table A 2.

**Table 1:** Study cohort characteristics, decisions on continued care after RRT-review and outcomes.

	**(n = 830)**
Age	72 (64, 80)
Female	356 (43)
**Clinical affiliation, n (%)**	
Medicine	304 (37)
Surgery	215 (26)
Orthopedic	80 (10)
Infection	68 (8)
Emergency Dept	39 (5)
Geriatric	16 (2)
Psychiatry	5 (1)
Other	103 (11)
**Symptoms/diagnosis at admission, n (%)**	
Surgical diseases	182 (22)
Infections	143 (18)
Orthopedic diseases	72 (9)
Sepsis	68 (8)
Dyspnoe	42 (5)
Maglinancy	27 (3)
Cardiovascular diseases	25 (3)
Respiratory diseases	20 (2)
Altered level of consciousness	20 (2)
Catastrophic conditions	20 (2)
Neurological diseases	15 (2)
Other cause of admission	196 (24)
**Primary reason for RRT review, n (%)**	
NEWS 2 score	489 (59)
Concern for the patient	213 (26)
Other causes	128 (15)
**Limitations of medical treatment prior to RRT review,** **n (%)**	108 (13)
DNACPR	97 (12)
Other	11 (1)
**NEWS 2 score median, (Q1, Q3)**	9 (6,11)
**Number of patients with NEWS 2 score** ≥ **7**	614 (74)
**Decision after RRT review, n (%)**	
Immediate transfer to the ICU	218 (26)
Patients transferred to HDU/CCU	91 (11)
Patients remaining at ward	521 (63)
**Patients receiving a new decision on Limitations of medical treatment**	67 (8)
**Outcomes, n (%)**	
ICU admission within 24 h of RRT-review	300 (36)
Cardiac arrest within 24 h of RRT-review	8 (1)
Mortality within 24 h of RRT-review	50 (6)
30-day mortality	257 (31)
90-day mortality	274 (33)

Data are presented as numbers (percentages), and continuous variables as medians with interquartile ranges (Q1, Q3), n=830.

RRT, Rapid Response Team, NEWS 2, National Early Warning Score 2, DNACPR, Do-not-attempt CPR, ICU, Intensive Care Unit, HDU, High Dependency Unit, CCU, Coronary Care Unit.

### Outcomes

The RRT-review resulted in an immediate transfer to the ICU in 218 cases (26 %). Within 24 h of RRT-review, a total of 300 patients (36 %) were admitted to the ICU. There was an escalation of the level of care in another 11 % of the patients, by transfer to a High Dependency Unit (HDU) or Coronary Care Unit (CCU) (Table 1). The 24-hour mortality was 6 %, and 1 % of the patients suffered from IHCA. In all, 331 patients (40 %) were affected by the composite outcome. The 30- and 90-day mortality was 31 % and 33 % respectively (Table 1).

**Fig. 2:**
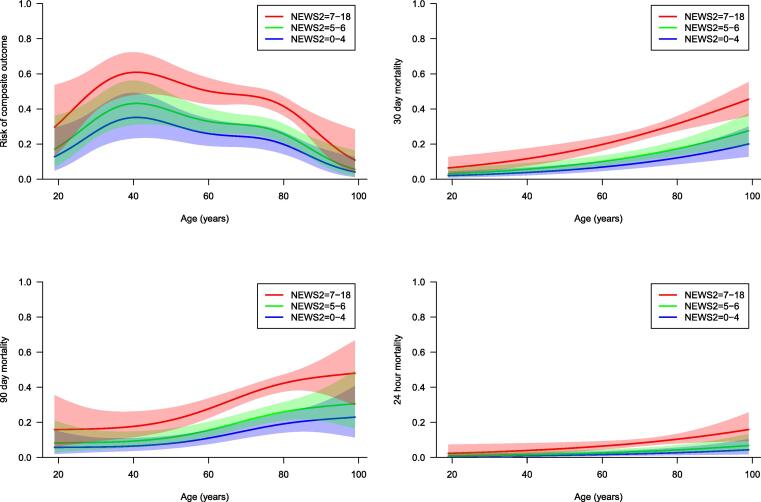
**The associations of age with the predicted outcomes**. The associations of age with the composite outcome (ICU admission, IHCA and mortality within 24 hours of RRT- review), 24-hour mortality, 30-day mortality and 90-day mortality in low, medium, and high-risk groups of patients by means of polynomial regression ( GAM with spline components for age and NEWS 2), with pointwise 95% confidence bands. ICU, Intensive care unit, RRT, Rapid Response Team, GAM, General Additive Models. A NEWS 2 score of 0-4 point is defined as low risk, 5-6 points as medium risk and ≥7 points as high risk.

The associations of age with the composite outcome, 24-hour mortality, 30-, and 90-day mortality in low, medium, and high-risk groups of patients, by means of polynomial regression (GAM with spline components for age and NEWS 2), with pointwise 95 % confidence bands, are presented in Fig. 2.

### The discriminative ability of NEWS 2

**Fig. 3:**
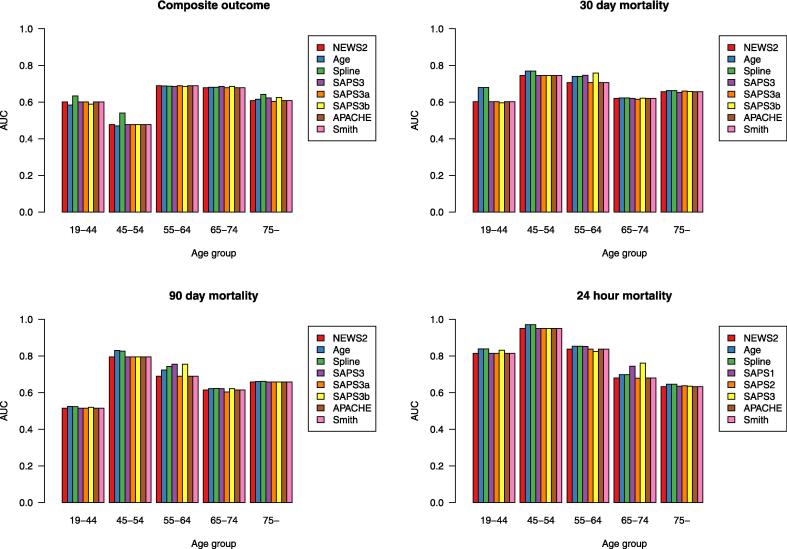
**The discriminative ability of NEWS 2 with and without age as a covariate**. The discriminative ability of NEWS 2 alone and with age as a continuous variable, in a spline regression model and categorized into different age models , to identify patients at risk of the composite outcome (unanticipated ICU admission, IHCA or mortality) within 24 hours of RRT-review and in prediction of 30- and 90-day mortality. NEWS 2, National Early Warning Score 2, ICU, Intensive Care Unit, IHCA, In-hospital cardiac arrest, RRT, Rapid Response Team, SAPS, Simplified Acute Physiology Score, APACHE, Acute Physiology, Age and Chronic Health Evaluation system. The model ”Smith” refers to the age categorizations by Smith et al. See Methods.

The AUROC of NEWS 2, without adding age, in predicting 24-hour and 30-day mortality among RRT-reviewed patients was 0.69 (0.65–0.76) and 0.66 (0.62–0.70) respectively (Table 2). Adding age as a covariate in prediction of 24-hour mortality did not improve the AUROC (Table 2, Figure A 2). Regarding prediction of 30-day mortality among RRT-reviewed patients, adding age improved the discriminative ability of NEWS 2 in all models (Table 2, Figure A 2). The best performances were observed using age as a continuous variable and the spline model, where the AUROC increased from 0.66 (0.62–0.70) to 0.70 (0.65–0.74, p = 0.01, Table 2). There appeared to be a difference in the discriminative ability of NEWS 2 according to age groups, with the apparent best predictions of mortality by adding age among patients aged 45–54 years (Fig. 3).

**Table 2:** The discriminative ability of NEWS 2 and in combination with the different age models.

	AUROC	(95 % CI)	p-value
ICU admission (<24 h)			
NEWS 2 without age	0.61	0.57–0.65	
Age as contiunous variable	0.63	0.59–0.65	0.06
NEWS 2_Spline	0.66	0.62–0.69	**<0.01**
NEWS 2_APACHE	0.64	0.60–0.68	**0.02**
NEWS 2_SAPS 3	0.64	0.60–0.68	**0.03**
NEWS 2_SAPS 3a	0.62	0.60–0.66	0.28
NEWS 2_SAPS 3b	0.64	0.60–0.68	**0.03**
NEWS 2_Smith	0.63	0.59–0.67	0.09
Mortality (<24 h)			
NEWS 2 without age	0.69	0.65–0.76	
Age as contiunous variable	0.72	0.65–0.78	0.15
NEWS 2_Spline	0.72	0.65–0.78	0.14
NEWS 2_APACHE	0.71	0.65–0.78	0.82
NEWS 2_SAPS 3	0.72	0.65–0.78	0.12
NEWS 2_SAPS 3a	0.70	0.64–0.77	0.43
NEWS 2_SAPS 3b	0.72	0.65–0.78	0.12
NEWS 2_Smith	0.71	0.64–0.78	0.25
IHCA (<24 h)			
NEWS 2 without age	0.54	0.32–0.76	
Age as contiunous variable	0.62	0.46–0.78	0.37
NEWS 2_Spline	0.62	0.46–0.78	0.37
NEWS 2_APACHE	0.61	0.42–0.79	**0.03**
NEWS 2_SAPS 3	0.65	0.48–0.82	0.06
NEWS 2_SAPS 3a	0.60	0.41–0.78	0.52
NEWS 2_SAPS 3b	0.67	0.49–0.84	**<0.01**
NEWS 2_Smith	0.61	0.42–0.78	**0.03**
Composite (<24 h)			
NEWS 2 without age	0.63	0.59–0.67	
Age as contiunous variable	0.64	0.60–0.68	**0.02**
NEWS 2_Spline	0.66	0.63–0.70	**0.01**
NEWS 2_APACHE	0.65	0.62–0.69	**0.02**
NEWS 2_SAPS 3	0.65	0.61–0.69	0.09
NEWS 2_SAPS 3a	0.64	0.60–0.68	0.28
NEWS 2_SAPS 3b	0.65	0.61–0.69	0.09
NEWS 2_Smith	0.64	0.62–0.68	0.10
30-day mortality			
NEWS 2 without age	0.66	0.62–0.70	
Age as contiunous variable	0.70	0.65–0.74	**0.01**
NEWS 2_Spline	0.70	0.65–0.74	**0.01**
NEWS 2_APACHE	0.69	0.65–0.73	**0.02**
NEWS 2_SAPS 3	0.69	0.65–0.73	**0.02**
NEWS 2_SAPS 3a	0.69	0.64–0.73	**0.03**
NEWS 2_SAPS 3b	0.69	0.65–0.73	**0.03**
NEWS 2_Smith	0.69	0.65–0.73	**0.02**
90-day mortality			
NEWS 2 without age	0.65	0.61–0.69	
Age as contiunous variable	0.68	0.64–0.72	**0.01**
NEWS 2_Spline	0.68	0.65–0.72	**0.01**
NEWS 2_APACHE	0.68	0.64–0.72	**0.01**
NEWS 2_SAPS 3	0.68	0.64–0.72	**0.01**
NEWS 2_SAPS 3a	0.67	0.63–0.71	0.05
NEWS 2_SAPS 3b	0.68	0.64–0.72	**0.01**
NEWS 2_Smith	0.68	0.64–0.71	**0.01**
			

The discriminative ability of NEWS 2 in combination with age as a continuous variable, in a spline regression model and categorized into different age models, in prediction of unanticipated ICU admission, IHCA or mortality, and the composite of these three, within 24 hours of RRT-review, and prediction of 30-, 90- and 180-day mortality, evaluated using AUROC.

AUROC, Area Under the Curve Receiver Operating Characteristics, NEWS 2, National Early Warning Score 2, ICU, Intensive Care Unit, IHCA, In-hospital cardiac arrest, RRT, Rapid Response Team, SAPS, Simplified Acute Physiology Score, APACHE, Acute Physiology, Age and Chronic Health Evaluation system. The age model ”Smith” refers to the age categorizations by Smith et al. See Methods.

The AUROC of NEWS 2, without age as a covariate, in predicting 90-day mortality was 0.65 (0.61–0.69, Table 2), improving to 0.68 (0.64–0.72, all models except the SAPS 3a, p = 0.01, Figure A 2) when adding age as a covariate (Table 2).

Regarding IHCA within 24 h of RRT review, the discriminative ability of NEWS 2 was improved by adding age as a covariate according to the APACHE, SAPS 3b and Smith models. The best performance was achieved using the SAPS 3b model, which increased the AUROC from 0.54 (0.32–0.76) to 0.67 (0.49–0.84, p = 0.003), as compared to NEWS 2 without adding age as a covariate (Table 2).

The AUROC for NEWS 2 in predicting unanticipated ICU admission within 24 h of RRT-review was 0.61 (0.57–0.65), improving according to the spline, APACHE, SAPS 3 and SAPS 3b models to at best 0.66 (0.62–0.69, p = 0.001, the spline model, Table 2).

Adding age as a covariate in prediction of the composite outcome improved the AUROC according to the continuous age, spline, and APACHE models from 0.63 (0.59–0.67) to at best 0.66 (0.63–0.70, the spline model, p = 0.01, Table 2, Figure A2).

The comparisons of Brier scores between NEWS 2 and NEWS 2 and age yields similar results as corresponding comparisons of AUROC, indicating that the models are well calibrated (Table 3).

**Table 3:** Brier scores.

Outcome	NEWS2	NEWS2 + Age	Difference	(95 % CI)	p-value
30-day mortality	0.1660	0.1613	0.0047	0.0009 – 0.0084	**<0.01**
90-day mortality	0.2016	0.1958	0.0058	0.0014 – 0.0100	**<0.01**
Composite	0.2269	0.2249	0.0020	−0.0011 – 0.0050	0.19
24-hour mortality	0.0688	0.0686	0.0002	−0.0011 – 0.0015	0.73
ICU admission	0.2231	0.2195	0.0036	−0.0004 – 0.0075	0.074
Cardiac arrest	0.00954	0.00952	0.00002	−0.00004 – 0.00010	0.64

CI, Confidence Interval, NEWS 2, National Early Warning Score 2, ICU, Intensive Care Unit.

## Discussion

This prospective, multi-centre study on a cohort of RRT-reviewed patients shows that adding age as a covariate to the NEWS 2 improves the discriminative ability to predict 30-day mortality to a level considered as acceptable. There appeared to be overall differences between patient age groups regarding the effect of adding age as a covariate to NEWS 2, with the best performance in prediction of mortality among patients aged 45–54 years. We also observed an improved discriminative ability in prediction of IHCA and unanticipated ICU-admission, as well as the composite outcome within 24 h of RRT-review, however remaining in a level of prediction considered as weak. Approximately two out of three RRT-reviewed patients were alive at 30-days. The discriminative ability of NEWS 2, with or without age as a covariate, in prediction of long-term prognosis was weak.

To the best of our knowledge, this is the first study exploring age as a covariate added to NEWS 2 in a cohort of RRT-reviewed patients. Our finding of an improved discriminative ability in prediction of 30-day mortality is most likely related to an increased rate of events as compared to 24-hour mortality.

Regarding the overall discriminative abilities, NEWS 2 was originally developed to predict significant adverse medical events in a general ward patient population, where NEWS 2 achieved excellent discriminative abilities in prediction of 24-hour mortality.^7^ In our current study, three out of four patients scored ≥ 7 points, which previously have been associated to a higher risk of significant adverse medical events.^17^ This has most likely inflicted outcomes and possibly underestimated the discriminative ability. We can only speculate if a large, general ward patient population would have generated overall higher discriminative abilities, when exploring the effect of age as a covariate to NEWS 2. This could be a useful topic for future research.

The differences across age groups in prediction of mortality is consistent with previous findings by Shamout et al., illuminating that accounting for age-related vital sign changes may lead to better model performance in patients aged 45 years or less.^25^ In another recent study on NEWS 2 and age, addition of age as a covariate improved overall prediction of in-hospital mortality, however an overestimation of risks for younger patients (aged 18 to 64 years) remained.^15^ Notably, these studies were not performed in a high-risk setting of patients, which clearly alters the prerequisites.

Many EWSs developed during the last decade are derived mathematically from actual clinical data, hence influenced by the patient case mix of the populations from which they are developed. In most cases, and certainly for the NEWS 2, the populations used to develop these “newer” scores comprised hospital inpatients, where the population is skewed towards older patients, which is also the case in this study cohort. For example, median age in the development set for ViEWS, subsequently modified into the NEWS, was 60 years.^26^ In a Finnish study on RRT-reviewed patients, every third patient was aged 75 years or more.^27^ Taken together, these previous findings might support our findings regarding the observed differences in discriminative abilities across different age categories, with a more beneficial effect among younger patients.

Few studies have described long-term prognosis among RRT-reviewed patients. Our findings of a 30-day mortality of 31 % extend the existing literature; in a systematic review, Tirkkonen et al. reported a median 30-day mortality rate of 29 % (8–39 %) among RRT-reviewed patients.^20^ Furthermore, our results underline the poor condition of RRT-reviewed patients, with variable and high mortality rates.^20^

Regarding prediction of IHCA, we found a poor discriminative ability of NEWS 2 alone. This is in line with the literature, and most likely reflects that IHCA is harder to predict and less common than other significant adverse medical events,^7,17^ due to sudden malignant arrythmias or coronary occlusions less commonly are heralded by a successive clinical deterioration accompanied by abnormalities in vital signs prior to CA. This notwithstanding, we observed a substantial improvement in prediction of IHCA by adding age as a covariate to NEWS 2, however remaining at a weak level of prediction.

In a recent study of RRT-reviewed patients, NEWS 2 alone did not improve the predictive performance in identifying patients at risk of significant adverse medical events within 24 h.^17^ Besides age related alterations in vital signs, measurements of biological capacity and function (e.g., frailty or mobility) need to be taken into consideration in prognostication.^28^ Previous studies have identified frailty and mobility as independent predictors of prognosis in old patients.^29^ However, incorporation of additional covariates like for example age and frailty might increase the complexity of the NEWS 2, resulting in a less user-friendly model. This notwithstanding, future NEWS 2 versions most likely need adaptation taking these variables in consideration and should probably be used in conjunction with other clinical assessments and variables. Given the technological advances over the last decades, where risk prediction models based on modern machine learning algorithms have shown to improve and outperform traditional EWS,^30^, ^31^, ^32^ a future option could be integrating NEWS 2 with digital, AI powered models taking variables like age and frailty into consideration. However, there will also most likely remain a need for the traditional NEWS 2 model in the future, above all in low-resource settings. Therefore, we need to continuously work on updating and improving NEWS 2, to achieve the best possible patient safety in the global perspective.

In summary, the aim of this study was not to construct a novel NEWS 2 scoring model including age as a variable, but rather explore the effect of adding age in a cohort of RRT reviewed patients. Our results could contribute and motivate to future studies on how to combine NEWS 2 with both age and variables assessing the biological capacity of the patient. Indicative of how age-related points in the score could be weighted, our study could serve as one possible piece of the decision-making jigsaw aiming to identify patients at higher risk following RRT-review.

## Strengths and limitations

Major strengths of this study include the prospective study design, covering 24 hospitals with varying demographic representation and geographic localization, the uniform, digital calculation of NEWS 2 scores, and complete information on the outcome by means of the Swedish Personal Identification Numbers and registries.^33^ Data collection in a real-world setting, where the RRT-reviews were performed according to clinical practice without adjustments, constitutes another strength.

However, there are also limitations. This study was conducted in a high-risk cohort of patients, hence very few patients were scoring low on the NEWS 2 scale. As previously stated, most RRT-reviews result in an intervention, possibly affecting outcome.^34^ In our study, the knowledge on specific RRT interventions was limited. Taken together, this might have inflicted the discriminative ability of NEWS 2. Furthermore, data collection did not include any information about comorbidities, frailty, or functional status. It is also possible that a Hawthorne effect arose due to the prospective study design.^35^

## Conclusion

Adding age as a covariate improved the discriminative ability of NEWS 2 in the prediction of 30-day mortality among RRT-reviewed patients, with variations observed across age categories. The long-term prognosis of RRT-reviewed patients was poor.

## CRediT authorship contribution statement

**Anna Thorén:** Conceptualization, Methodology, Formal analysis, Investigation, Writing – original draft, Writing – review & editing, Visualization, Submission of manuscript, Funding acquisition. **Mikael Andersson Franko:** Conceptualization, Methodology, Investigation, Formal analysis, Writing – review & editing, Visualization. **Eva Joelsson-Alm:** Conceptualization, Methodology, Writing – review & editing. **Araz Rawshani:** Conceptualization, Methodology, Writing – review & editing. **Thomas Kahan:** Conceptualization, Methodology, Writing – review & editing. **Johan Engdahl:** Conceptualization, Methodology, Writing – review & editing. **Martin Jonsson:** Conceptualization, Methodology, Investigation, Writing – review & editing. **Therese Djärv:** Conceptualization, Methodology, Writing – review & editing. **Martin Spångfors:** Conceptualization, Methodology, Investigation, Formal analysis, Writing – review & editing, Visualization, Study supervision.

## Declaration of competing interest

The authors declare that they have no known competing financial interests or personal relationships that could have appeared to influence the work reported in this paper.
