# Supplementary material for: Exploring the impact of age on the predictive power of the National Early Warning score (NEWS) 2, and long-term prognosis among patients reviewed by a Rapid Response Team: A prospective, multi-centre study
Source: Resusc Plus. 2024 Dec 10;21:100839. doi: 10.1016/j.resplu.2024.100839 (PMC11732060; doi:10.1016/j.resplu.2024.100839)
Supplement: Supplementary Data 1 [file mmc1.docx]

| Table A 1  Participating hospitals (n=24); demographics, type of EWS, number of beds overall/ in the ICU, HDU availability, RRT availability, number of RRT assessments and number of included patients during the study period. | | | | | | | | |
| --- | --- | --- | --- | --- | --- | --- | --- | --- |
|  | Hospital category | EWS | # Beds | # ICU Beds | HDU (Y/N) | RRT 24/7 (Y/N) | # RRT | # Included patients |
|  | **Total** |  | **7,689** | **202** |  |  | **1,208** | **998** |
| 1 | University | NEWS 2 | 571 | 16 | Yes | Yes | 119 | 106 |
| 2 | University | NEWS | 560 | 8 | Yes | No | 125 | 30 |
| 3 | University | NEWS 2 | 526 | 20 | Yes | Yes | 160 | 97 |
| 4 | University | NEWS 2 | 500 | 8 | Yes | Yes | 26 | 24 |
| 5 | University | NEWS 2 | 490 | 21 | Yes | Yes | 48 | 43 |
| 6 | University | NEWS | 473 | 9 | Yes | Yes | 53 | 35 |
| 7 | University | NEWS | 470 | 10 | Yes | Yes | 115 | 96 |
| 8 | University | NEWS | 439 | 10 | No | Yes | 21 | 15 |
| 9 | University | NEWS, ** | 330 | 8 | Yes | Yes | 29 | 22 |
| 10 | University | NEWS 2 | 294 | 5 | Yes | Yes | 45 | 38 |
| 11 | County | NEWS | 453 | 12 | Yes | Yes | 148 | 102 |
| 12 | County | NEWS 2 | 339 | 8 | Yes | Yes | 34 | 30 |
| 13 | County | NEWS 2 | 300 | 6 | No | Yes | 17 | 16 |
| 14 | County | NEWS | 250 | 6 | No | Yes | 42 | 41 |
| 15 | County | NEWS | 240 | 7 | No | Yes | 41 | 30 |
| 16 | County | NEWS 2 | 220 | 6 | No | Yes | 38 | 35 |
| 17 | County | NEWS 2 | 215 | 11 | No | Yes | 35 | 31 |
| 18 | County | NEWS 2 | 200 | 6 | No | Yes | 31 | 27 |
| 19 | County | NEWS 2 | 184 | 6 | Yes | Yes | 36 | 35 |
| 20 | District | NEWS, * | 317 | 6 | No | Yes | 9 | 8 |
| 21 | District | NEWS 2 | 110 | 4 | No | Yes | 21 | 20 |
| 22 | District | NEWS 2 | 107 | 3 | Yes | Yes | 4 | 4 |
| 23 | District | NEWS 2 | 60 | 3 | No | Yes | 6 | 6 |
| 24 | District | NEWS 2 | 41 | 3 | No | Yes | 5 | 5 |

| * NEWS 2 from January 1, 2020 |
| --- |
| ** NEWS 2 from January 21, 2020 |

NEWS, Early warning score, ICU, Intensive Care Unit, HDU, High Dependency Unit, RRT, Rapid Response Team.

**Table A 2 - The different age models and NEWS 2 scores**Data are presented as medians with interquartile ranges (Q1, Q3).

| SAPS 3 | | SAPS 3a | | SAPS 3b | |
| --- | --- | --- | --- | --- | --- |
| Age group | **NEWS 2** | **Age group** | **NEWS 2** | **Age group** | **NEWS 2** |
| 18-59 years | 8 (6 – 10) | 18-65 years | 8 (6 – 10) | 18-39 years | 8 (6 – 10) |
| 60-69 years | 8.5 (6 – 10) | 66-75 years | 8 (6 – 11) | 40-59 years | 8 (6 – 10) |
| 70-79 years | 9 (7 – 11) | 76- years | 9 (7 – 11) | 60-69 years | 9 (6 – 10) |
| 80- years | 9 (7 – 11) |  |  | 70-74 years | 9 (7 – 11) |
|  |  |  |  | 75-79 years | 9 (7 – 11) |
|  |  |  |  | 80- years | 9 (7 – 11) |

| APACHE | | Smith | |
| --- | --- | --- | --- |
| Age group | **NEWS 2** | **Age group** | **NEWS 2** |
| 18-44 years | 8 (6 – 10) | 18-39 years | 8 (6 – 10) |
| 45-54 years | 8.5 (6 – 10) | 40-64 years | 8 (6 – 10) |
| 55-64 years | 9 (7 – 11) | 65-79 years | 9 (6 – 10) |
| 65-74 years | 9 (7 – 11) | 80- years | 9 (7 – 11) |
| 75- years | 9 (7-11) |  |  |
|  |  |  |  |

NEWS 2, National Early Warning Score 2, SAPS, Simplified Acute Physiology Score, APACHE, Acute Physiology, Age and Chronic Health Evaluation system.
The age model ”Smith” refers to the age categorizations by Smith et al. See Methods.

**Table A 3- Symptoms/diagnosis at admission in detail (n=830).**

Data are presented as numbers (percentages and cumulative sum).

|  | | | |
| --- | --- | --- | --- |
| Variable | Value (n=830) |  |  |
| Surgical diseases | 127 | 15.3 |  |
| Other infections | 77 | 9.2 |  |
| Orthopedic diseases | 72 | 8.6 |  |
| Pneumonia and respiratory infections | 67 | 8.0 |  |
| Sepsis | 67 | 8.0 |  |
| Other cause of admission | 56 | 6.7 |  |
| Dyspnoe | 42 | 5.0 |  |
| Abdominal pain | 39 | 4.6 |  |
| Malignancy | 27 | 3.2 |  |
| Cardiovascular diseases | 25 | 3,0 |  |
| Respiratory diseases | 20 | 2.4 |  |
| Altered level of consciousness | 20 | 2.4 |  |
| Catastrophic conditions | 20 | 2.4 |  |
| Gastrointestinal bleeding | 16 | 1.9 |  |
| Neurological diseases | 15 | 1.8 |  |
| Impaired general condition | 13 | 1.5 |  |
| Intoxication | 13 | 1.5 |  |
| Gastrointestinal diseases | 11 | 1.3 |  |
| Malignancy and infection in combination | 11 | 1.3 |  |
| Chest pain | 10 | 1.2 |  |
| Trauma | 10 | 1.2 |  |
| Respiratory diseases and infection | 10 | 1.2 |  |
| Psychiatric diseases | 8 | 0.9 |  |
| Haematological diseases | 8 | 0.9 |  |
| Renal diseases | 7 | 0.8 |  |
| Diabetic emergencies | 5 | 0.6 |  |
| Metabolic or endocrine diseases. electrolytes | 5 | 0.6 |  |
| Ascites | 6 | 0.7 |  |
| Respiratory and cardiovascular diseases in combination | 3 | 0.4 |  |
| Tromboembolic diseases | 3 | 0.4 |  |
| Ophtalmological diseases | 3 | 0.4 |  |
| Syncope | 2 | 0.2 |  |
| Rheumatological diseases | 2 | 0.2 |  |
| Gynecological diseases | 2 | 0.2 |  |
| Swollen legs | 1 | 0.1 |  |
| Missing data | 7 | 0.8 |  |

Catastrophic conditions include aortic aneurysm (ruptured), aortic dissection, circulatory failure, cardiac arrest, cardiogenic shock, hypertensive crisis, hypothermia, intracerebral haemorrhage, multiple organ failure and suicide (hanging).

**Table A 4- Primary reason for RRT-review, a detailed description of the category “other” (n= 128)**

Data are presented as numbers (percentages).

|  | |
| --- | --- |
|  |  |
| Respiratory distress | 40 (31) |
| Circulatory distress | 36 (28) |
| RRT follow- up | 11 (9) |
| Mental status change | 10 (8) |
| Blood test result abnormality | 7 (5) |
| Altered diuresis | 6 (5) |
| Assessment need for intensive care | 4 (3) |
| Assessment need of dialysis | 2 (1) |
| Other | 12 (10) |

Other cause include pain without further specification, gastrointestinal bleeding, bleeding without further specification, abdominal/chest, severe back pain, sepsis/suspected sepsis, epileptic seizure, arterial catheterization, discussion of level of care and preoperative assessment.

RRT, Rapid Response Team.

**Figure A 1 – The age distribution in the study cohort.**

**Figure A 2- AUROC curves for prediction of the composite outcome (unanticipated ICU admission, mortality or IHCA within 24 hours of RRT-review), 24-hour, 30-day, and 90-day mortality.**

Green = AUROC for NEWS 2 alone, Red= NEWS 2 and SAPS 3, Blue= NEWS 2 and SAPS 3b.

AUROC, Area Under the Curve Receiver Operating Characteristics, ICU, Intensive Care Unit, IHCA, In-hospital cardiac arrest, NEWS 2, National Early Warning Score 2, SAPS, Simplified Acute Physiology Score.
